# Comparative Proteomic Analysis of the PhoP Regulon in *Salmonella enterica* Serovar Typhi Versus Typhimurium

**DOI:** 10.1371/journal.pone.0006994

**Published:** 2009-09-10

**Authors:** Richelle C. Charles, Jason B. Harris, Michael R. Chase, Lauren M. Lebrun, Alaullah Sheikh, Regina C. LaRocque, Tanya Logvinenko, Sean M. Rollins, Abdullah Tarique, Elizabeth L. Hohmann, Ian Rosenberg, Bryan Krastins, David A. Sarracino, Firdausi Qadri, Stephen B. Calderwood, Edward T. Ryan

**Affiliations:** 1 Division of Infectious Diseases, Massachusetts General Hospital, Boston, Massachusetts, United States of America; 2 Department of Medicine, Harvard Medical School, Boston, Massachusetts, United States of America; 3 Department of Pediatrics, Harvard Medical School, Boston, Massachusetts, United States of America; 4 Harvard Medical School–Partners Healthcare Center for Genetics and Genomics, Cambridge, Massachusetts, United States of America; 5 Department of Immunology and Infectious Diseases, Harvard School of Public Health, Boston, Massachusetts, United States of America; 6 International Centre for Diarrhoeal Disease Research, Bangladesh, Dhaka, Bangladesh; 7 Biostatistics Research Center, Tufts Medical Center, Boston, Massachusetts, United States of America; 8 Department of Microbiology and Molecular Genetics, Harvard Medical School, Boston, Massachusetts, United States of America; University of Hyderabad, India

## Abstract

**Background:**

*S.* Typhi, a human-restricted *Salmonella enterica* serovar, causes a systemic intracellular infection in humans (typhoid fever). In comparison, *S.* Typhimurium causes gastroenteritis in humans, but causes a systemic typhoidal illness in mice. The PhoP regulon is a well studied two component (PhoP/Q) coordinately regulated network of genes whose expression is required for intracellular survival of *S. enterica*.

**Methodology/Principal Findings:**

Using high performance liquid chromatography mass spectrometry (HPLC-MS/MS), we examined the protein expression profiles of three sequenced *S. enterica* strains: *S.* Typhimurium LT2, *S.* Typhi CT18, and *S.* Typhi Ty2 in PhoP-inducing and non-inducing conditions *in vitro* and compared these results to profiles of *phoP^−^/Q^−^* mutants derived from *S.* Typhimurium LT2 and *S.* Typhi Ty2. Our analysis identified 53 proteins in *S.* Typhimurium LT2 and 56 proteins in *S.* Typhi that were regulated in a PhoP-dependent manner. As expected, many proteins identified in *S.* Typhi demonstrated concordant differential expression with a homologous protein in *S.* Typhimurium. However, three proteins (HlyE, STY1499, and CdtB) had no homolog in *S.* Typhimurium. HlyE is a pore-forming toxin. STY1499 encodes a stably expressed protein of unknown function transcribed in the same operon as HlyE. CdtB is a cytolethal distending toxin associated with DNA damage, cell cycle arrest, and cellular distension. Gene expression studies confirmed up-regulation of mRNA of HlyE, STY1499, and CdtB in *S.* Typhi in PhoP-inducing conditions.

**Conclusions/Significance:**

This study is the first protein expression study of the PhoP virulence associated regulon using strains of *Salmonella* mutant in PhoP, has identified three Typhi-unique proteins (CdtB, HlyE and STY1499) that are not present in the genome of the wide host-range Typhimurium, and includes the first protein expression profiling of a live attenuated bacterial vaccine studied in humans (Ty800).

## Introduction

*Salmonella enterica* serovar Typhi (*S.* Typhi) infects an estimated 22 million individuals each year, resulting in approximately 200,000 deaths [1]. *S.* Typhi is a facultative intracellular pathogen spread through contaminated food and water. In contrast to most other *Salmonella enterica* serovars, *S.* Typhi is human restricted, causing an invasive systemic illness in humans (typhoid fever) characterized by persistent fevers, abdominal pain, hepatosplenomegaly, and a myriad of complications including encephalopathy and intestinal perforation or hemorrhage [2]. Since *S.* Typhi does not cause a typhoidal illness in animals, most of what is known about the pathogenesis of *S.* Typhi has been extrapolated from analysis of *Salmonella enterica* serovar Typhimurium (*S.* Typhimurium), a wide-host range organism that does cause a disseminated illness in mice, but usually gastroenteritis in humans. Comparative genomic studies demonstrate significant differences between these two serovars, with approximately 11–13% of open reading frames (ORFs) being unique to *S.* Typhimurium compared with *S.* Typhi, and vice versa [3].

**Figure 1:**
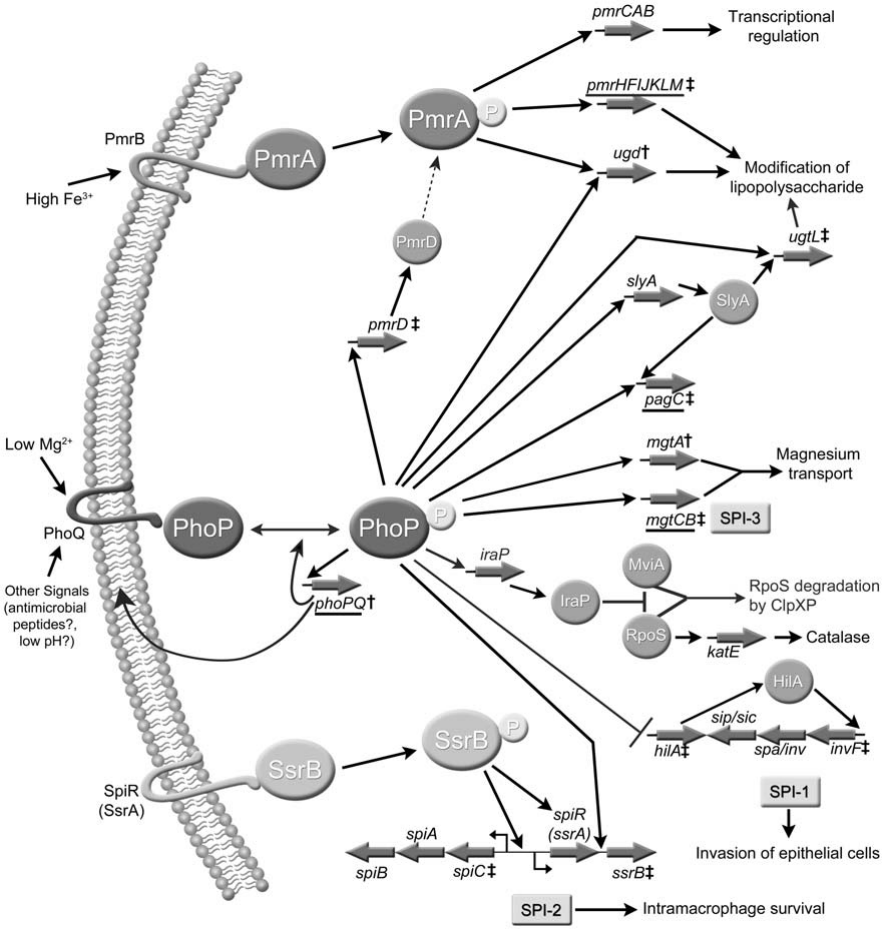
Illustration of the PhoQ/PhoP two-component regulatory system in *Salmonella enterica* serovar Typhimurium. PhoQ activates PhoP in response to a number of environmental signals including low magnesium. Once activated, PhoP can directly activate its own transcription and the transcription of a number of other genes. A number of directly regulated gene products then regulate additional regulatory cascades, including PmrD, which is able to activate the PmrAB operon independent of iron; SlyA, which regulates genes important to intra-macrophage survival such as *pagC* and *ugtL*; IraP which prevents MviA-dependent degradation of RpoS leading to RpoS accumulation and its regulation of genes important for stationary phase survival and resistance to oxidative stress; HilA, which is an inducer of SPI-1 (*Salmonella* pathogenicity island-1), which contains genes involved in invasion of epithelial cells; and SsrB, which is an inducer of SPI-2 containing genes important in intra-macrophage survival (adapted from Groisman E. and Mouslim C. *Nature Reviews Microbiology* (2006) 4∶705–709) [44], [45]. In this figure, underlined genes denote those whose products were detected in our analysis. †: Promoter region contains a typical PhoP box defined as a dyad of (T/G)GTTTA separated by 5 nucleotides. ‡: Presence of an atypical PhoP box defined as a dyad of (T/G)GTTTA separated by 5 nucleotides in the promoter region, allowing four substitutions as long as the following positions were conserved: a thymine in the first dyad half (at position 3) and two conserved thymines and one conserved adenine in the second dyad half at positions 3, 4, and 6, respectively, within 300 nucleotides of the translational start site (see text).

Despite such differences, *phoP* and *phoQ* are present in both *S.* Typhi and *S*. Typhimurium. PhoP/Q is a two-component regulatory system that controls expression of a network of genes involved in virulence and survival of several Gram negative pathogens, including *Shigella flexerni*, *Yersinia pestis*, and *Salmonella enterica* [4]. Interestingly, many PhoP/Q-regulated genes are species specific, conferring unique phenotypes [4]. In *S.* Typhi and *S.* Typhimurium, PhoP/Q is required for intra-macrophage survival, and mutations in *phoP/Q* result in a marked decrease in virulence [5]–[7]. In response to a number of environmental signals, including low magnesium, cationic peptides, and antimicrobial peptides (which may reflect conditions within vacuoles of macrophages), PhoQ activates PhoP, which then regulates expression of genes containing a “PhoP box” within promoters [8]–[14]. Expression of such directly regulated genes may then regulate additional regulatory cascades, including those controlled through Mig-14, SlyA, PmrD, and RpoS [11], [15]–[19] (Figure 1).

Previous application of high throughput genomic and proteomic technologies have given insight into transcriptome and proteomic profiles of *Salmonella* grown in conditions selected to mimic in vivo conditions, as well as using *Salmonella* harvested from macrophages [9], [20]–[27]. These studies have given important insights into expression profiles of *Salmonella* grown in conditions thought to induce the PhoP regulon as well as other regulatory cascades activated in vivo; however, these studies did not include strains mutant in PhoP, complicating the ability to associate results with the PhoP regulon specifically. To address this and to gain an improved understanding of PhoP/Q-dependent protein expression differences between *S.* Typhi and *S.* Typhimurium, we performed comparative proteomic analysis of PhoP/Q-regulated proteins in *S.* Typhimurium strain LT2, *S.* Typhi strain Ty2, and *S.* Typhi strain CT18, as well as *phoP^−^/Q* ^−^ mutant strains for LT2 and Ty2 (Ty800, a mutant strain being evaluated as a defined candidate typhoid vaccine in humans).

## Materials and Methods

### Bacterial strains, media, and growth conditions

Wild type *S.* Typhimurium LT2, *S.* Typhi Ty2, and *S.* Typhi CT18 were obtained from the Salmonella Genetic Stock Centre, University of Calgary, Calgary, Alberta, Canada. *S.* Typhi strain Ty800 is a previously described *phoP^−^/Q^−^* mutant of Ty2 [28], [29]. LT2 *phoP^−^/Q^−^* is a mutant of LT2 generated using a P22 bacteriophage lysate derived from *phoP^−^/Q^−^* mutant CS015 [*phoP102*: Tn*10d-Cam*] [29], [30]. To prepare bacteria for analysis, we grew strains in M9 minimal media (MM) containing either 10 mM MgCl_2_ (high Mg) or 10 µM MgCl_2_ (low Mg/PhoP-inducing condition) to mid-log phase. To confirm PhoP induction, we assayed an aliquot of culture for PhoN phosphatase activity using a BCIP (5-bromo-4-chloro-3′-indolyphosphate p-toluidine salt) assay modified from Kier et. al and Behlau et. al [31], [32].

### Sample preparation

To prepare samples for proteomic analysis, we re-suspended cell pellets in 8 M guanidine-HCL, 5% N-propanol, 100 mM ammonium bicarbonate, 10 mM dithiothreitol (DTT), and lysed cells using 0.1 mM silica beads (MP Biomedical, Solon, OH) and a Mini-Bead-Beater-8™ (BioSpec Products, Bartlesville, OK). Samples were then sonicated, boiled, cooled, alkylated with iodoacetic acid (dissolved in 1 M NH_4_HCO_3_ pH 8.5), quenched with excess DTT, dialyzed using 3,500 MWCO dialysis cassettes (Thermo Scientific, Rockford, IL) against Milli-Q water, then against 50 mM Tris and 2 mM MgCl_2_ pH 8.0 with benzonase, and then re-dialyzed against Milli-Q water. Samples were removed, frozen, and lyophilized. Lyophilized samples were re-dissolved in 8 M urea, 1% sodium dodecyl sulfate (SDS), 100 mM NH_4_HCO_3_, 10 mM DTT, and 1X LDS Loading Buffer (Invitrogen, Carlsbad, CA), pH 8.5. Samples were then heated, centrifuged to pellet insoluble protein, and fractionated on a one dimensional 10% Bis Tris NuPAGE MOPS gel (Invitrogen). Gels were then fixed in destain (50% methanol and 7.5% acetic acid), rehydrated, stained with Simply Blue Safestain (Invitrogen), cut horizontally into slices, and destained until transparent. Gel samples were rinsed with three alternating washes of 50 mM ammonium bicarbonate and acetonitrile. Samples were cooled to 4°C and subsequently each gel slice was resuspended in trypsin (5.5 µg/mL in 50 mM ammonium bicarbonate/10% acetonitrile) and incubated at 37°C for 24 hours for digestion of proteins. Peptides were extracted with one rinse of 50 mM ammonium bicarbonate/10% acetonitrile followed by one rinse of 50% acetonitrile/0.1% formic acid. Samples were prepared for mass spectrometry by lyophilization and rehydration in 100 µL 5% acetonitrile/0.1% formic acid.

### LC-MS analysis

Samples were loaded into 96-well plates for mass spectrometry analysis on an LTQ XL (Thermo Fisher Scientific) instrument. For each run, we injected 10 µL of each re-constituted sample using a Famos Autosampler (LC Packings). Reverse phase chromatographic separation was performed using Magic C18AQ 200A (Michrom, Auburn, CA), 5 µm packed into a fused silica 75 µm inner diameter, 15 cm long column (Polymicro Technologies, Phoenix, AZ) running at 250 nL/min from a Surveyor MS pump with a flow splitter. A gradient was produced between 5–40% acetonitrile, 0.35% formic acid over 90 minutes. The LTQ XL was run in a top eight configuration with one MS scan and eight MS/MS scans, with dynamic exclusion extending over 30 seconds.

### Peptide identification and statistical analysis

Peptide identifications were made using SEQUEST (Thermo Fisher Scientific) through Bioworks Browser, version 3.2. MS/MS data were searched using a 2 Da window on precursor m/z and a 1 Da window on fragment ions. Fully enzymatic tryptic searches with up to three missed cleavage sites were allowed. Oxidized methionines were searched as a variable modification and alkylated cysteines were searched as a fixed modification. Salmonella databases for LT2, CT18 and Ty2 were downloaded from Swiss-Prot and supplemented with common contaminants. A reverse database strategy [33] was employed by concatenating reversed protein sequences for each database entry in SEQUEST. Peptides for each charge state were filtered to a false discovery rate (FDR) of 1%, and peptides were then grouped into proteins using Occam's razor logic. Spectral counting was used to compare relative changes in protein abundance across two growth conditions and proteins required at least 3 spectral counts to be included in our analyses. We used a G-test with Benjamini-Hochberg correction to test for significant differential protein expression [34], and categorized a protein as PhoP-dependent if we detected significant differences in protein expression in wild type strains grown in PhoP-inducing versus non-inducing conditions following multiple comparison correction, and no significant differences in *phoP-/Q-* strains grown in PhoP-inducing versus non-inducing conditions. To minimize the likelihood of falsely categorizing an identified protein as PhoP-regulated in one strain but not another, once we identified a protein to be significantly regulated in any strain following multiple comparison correction, we considered it significantly regulated in any other strain with an uncorrected p value of ≤0.2. We identified homologous proteins using DAGchainer (http://dagchainer.sourceforge.net/) [35].

### RNA isolation, probe labeling, array hybridization, and genomic analysis

To analyze expression of genes associated with identified proteins, we added TRIzol (Life Technologies) to cultures grown in PhoP-inducing and non-inducing conditions. Procedure for DNase treatment of samples, construction of fluorescent cDNA, and hybridization to arrays were carried out as previously described with minor modifications [36]. Samples from the same strain grown in PhoP-inducing and non-inducing conditions were differentially labeled with Cy-3 and Cy-5 and hybridized to *Salmonella* Typhimurium/Typhi microarrays (version 4) consisting of 5,462 oligonucleotides encompassing open reading frames of *S.* Typhimurium LT2 and *S.* Typhi CT18 and Ty2 provided by the Pathogen Functional Genome Resource Center (PFGRC). Arrays were scanned using a ScanArray Express Instrument (PerkinElmer Life Sciences) and signal intensities quantified using ScanArray Express software, version 4 (Perkin Elmer). Following normalization, we analyzed intensities for *cdtB*, *hlyE*, STY1499, *pltA*, *pltB*, and *slyA* using log-transformed data fitting a mixed linear model (condition and dye) and slide as the random effect. All data are MIAME compliant and the raw data has been deposited at GEO (accession number: GSE17670).

### Motif detection

We used Motif Matcher (http://www.soe.ucsc.edu/~kent/improbizer/motifMatcher.html) to identify potential PhoP boxes in genomic sequences.

### Data deposition

The data associated with this manuscript (including raw spectra, spectral counts, and statistical values) are available either in the associated supplemental material (Table S1, Table S2, Table S3) or will be freely and publicly available at https://proteomecommons.org/tranche (accession: PhoPSalmonella032509) and GEO at www.ncbi.nlm.nih.gov/geo (accession number: GSE17670).

## Results

Using high performance liquid chromatography mass spectrometry (HPLC-MS/MS), we examined the proteome of *S.* Typhimurium LT2, *S.* Typhi CT18, and *S.* Typhi Ty2 grown to mid-log phase in M9 minimal media (MM) containing either 10 mM MgCl_2_ (high Mg) or 10 µM MgCl_2_ (low Mg/PhoP-inducing condition). Similarly, we examined the proteomic profile of *phoP^−^/Q^−^* mutants derived from *S.* Typhimurium LT2 and *S.* Typhi Ty2. We identified 1,071 proteins in *S.* Typhimurium LT2, 1,013 in *S.* Typhi CT18, and 1,179 in Ty2. We identified 44 PhoP-regulated proteins in *S.* Typhi CT18, 46 in *S.* Typhi Ty2, and 53 in *S.* Typhimurium LT2; in total, we identified 67 unique PhoP-regulated proteins in our analysis (Table S1). These proteins can be categorized into a number of functional groups (Table 1). Sixteen of the identified PhoP-regulated proteins have previously been associated with PhoP regulation, including PhoP, PhoN, PagC, SlyB, Udg, ArnB (PmrH), and VirK [4], [9], [37]. Forty-three of the identified PhoP-regulated proteins were common to both *S.* Typhi and *S.* Typhimurium (Table S2 and Table S3). We identified eleven in *S*. Typhimurium and not *S*. Typhi (Table 2). Two of them, pSLT046 and DkgA, lacked corresponding functional gene sequences in *S.* Typhi. Of the remaining nine, MgtB has previously been shown to be PhoP-dependent using genomic analyses [4], [9].

**Table 1:** Functional categories of detected PhoP-regulated proteins.

Classification	*S*. Typhi	*S.* Typhimurium
Pathogenicity/adaptation/chaperones	6	5
Regulators	4	4
Membrane/surface structures	11	11
Central/intermediary/miscellaneous metabolism	16	13
Phage/IS elements	2	2
Energy metabolism	3	3
Degradation of small and macromolecules	1	3
Information transfer	11	10
Conserved hypothetical	1	1
Unknown	1	1
Total	56	53

**Table 2:** PhoP-dependent proteins identified in our proteomic analysis in *S.* Typhimurium and not in *S.* Typhi*.

CT 18 Locus	Ty2 Locus	LT2 Locus	Gene Name	Function
		PSTL046	§ ‡	putative carbonic anhydrase
		STM3165	*dkgA* §	2,5 diketo-D-gluconic acid reductase A
STY4023	t3755	STM3763	*mgtB*	Mg^2+^ transporter
STY4911	t4604	STM4561	*osmY* ‡	hyperosmotically-inducible periplasmic protein
STY2493	t0597	STM2267	*ompC* ‡	outer membrane protein C precusor
STY3926	t3666	STM3857	*pstS* ‡	high-affinity phosphate transporter
STY3929	t3669	STM3854	*pstB*	phosphate ABC transporter, ATP-binding protein
STY2710	t0257	STM2473	*talA*	transaldolase
STY0780	t2139	STM0737	*sucB* **	2-oxoglutarate dehydrogenase, E2 component, dihydrolipoamide succinyltransferase
STY3930	t3670	STM3853	*phoU*	phosphate transport system regulatory protein
STY0231	t0210	STM0209	*htrA*	Heat shock protein

* These proteins were either not detected in our proteomic analysis of *S.* Typhi; or if detected, significant differential expression was not observed between PhoP-inducing and non-inducing condition, or if present, this regulation was not found to be PhoP-dependent.

** Repressed.

§ Unique to genome of *S.* Typhimurium.

‡ Presence of an atypical PhoP box defined as a dyad of (T/G)GTTTA separated by 5 nucleotides in the promoter region, allowing four substitutions as long as the following positions were conserved: a thymine in the first dyad half (at position 3) and two conserved thymines and one conserved adenine in the second dyad half at positions 3, 4, and 6, respectively, within 200–300 nucleotides of transcriptional start site (see text).

We identified fourteen PhoP-regulated proteins in *S*. Typhi and not in S. Typhimurium, of which three lacked corresponding gene sequences in *S.* Typhimurium: cytolethal distending toxin B (CdtB), hemolysin E (HlyE), and STY1499, a protein possibly involved in invasion of macrophages [38] (Table 3). We evaluated genomic expression of these proteins in PhoP-inducing compared to non-inducing conditions and found that the corresponding mRNA was significantly elevated for *S.* Typhi strain Ty2 (HlyE: 87-median fold increase [80, 119; quartiles], p<0.026; STY1499: 107-fold increase [89, 139; quartiles], p<0.023) and for strain CT18 (CdtB: 510-fold increase [365, 775; quartiles], p<0.021; HlyE: 71-fold increase [42, 100; quartiles], p<0.012; and STY1499: 86-fold increase [59, 128; quartiles], p<0.029). There was no significant increase of mRNA detected for any of these genes in S. Typhi *phoP^−^/Q^−^* mutant strain Ty800. mRNA was also significantly elevated in PhoP-inducing conditions compared to non-inducing conditions for CdtB-associated proteins PltA (80-fold increase [60, 92; quartiles], p<0.044) and PltB (80-fold increase [74, 93; quartiles], p<0.029) in *S.* Typhi strain CT18. SlyA was also found to be significantly elevated in PhoP-inducing compared to non-inducing conditions for *S*. Typhi strain Ty2 (5.1 fold increase [4.8, 7.6; quartiles], p<0.048) and for strain CT18 (7-fold increase [6.6, 8.6; quartiles], p<0.036).

**Table 3:** PhoP-dependent proteins identified in our proteomic analysis in *S.* Typhi and not in *S.* Typhimurium*.

Ct18 Locus	Ty2 Locus	LT2 Locus	Gene Name	Function
STY1498	t1477		*hlyE* § ‡	haemolysin HlyE
STY1499	t1476		§	conserved hypothetical protein
STY1886	t1111		*cdtB* § ‡	putative toxin-like protein
STY0440	t2461	STM0402		antioxidant, AhpC/Tsa family, authentic frameshift
STY3094	t2865	STM2956	*relA* ‡	GTP pyrophosphokinase
STY0937	t1992	STM0941	*ybjY* †	probable exported protein
STY2288	t0794	STM2079	*cld* ‡	polysaccharide chain length regulator
STY3909	t3650	STM3869	*atpF*	ATP synthase F0, B subunit
STY3881	t3621	STM3999	*polA* ‡	DNA polymerase I
STY3243	t3002	STM3090	*metK* ** ‡	S-adenosylmethionine synthetase
STY2711	t0385	STM2474	*tktB*	transketolase
STY3938	t3678	STM3842	*yidC* **	probable membrane protein
STY2802	t0301	STM2555	*glyA* **	serine hydroxymethyltransferase
STY3648	t3389	STM3909	*ilvC* **	ketol-acid reductoisomerase

* These proteins were either not detected in our proteomic analysis of *S.* Typhimurium; or if detected, significant differential expression was not observed between PhoP-inducing and non-inducing condition, or if present, this regulation was not found to be PhoP-dependent.

** Repressed.

§ Unique to Genome of *S.* Typhi.

† Promoter region contains a typical PhoP box defined as a dyad of (T/G)GTTTA separated by 5 nucleotides.

‡ Presence of an atypical PhoP box defined as a dyad of (T/G)GTTTA separated by 5 nucleotides in the promoter region, allowing four substitutions as long as the following positions were conserved: a thymine in the first dyad half (at position 3) and two conserved thymines and one conserved adenine in the second dyad half at positions 3, 4, and 6, respectively, within 200–300 nucleotides of transcriptional start site (see text).

## Discussion

We generated protein expression profiles to compare the PhoP regulon of *S.* Typhi to *S.* Typhimurium and identified 67 PhoP-dependent proteins in our analysis. Sixteen have previously been associated with the PhoP system, supporting the validity of our approach [4], [9], [37]. Gene regulation in the PhoP regulon is thought to occur through either direct binding of PhoP at a “PhoP box” in the promoter region of a gene, or through secondary cascade effects of a directly PhoP-regulated protein. The genes for a number of the PhoP-regulated proteins detected in this study contain a “classic PhoP box” (a direct repeat of six nucleotides, [T/G]GTTTA, separated by 5 nucleotides), including PhoP itself, YbjY, and Udg [10], [39]–[41]. Recent data suggest that PhoP is also able to bind to promoter regions without this exact motif, as long as a conserved thymine is preserved in the first half of the dyad (at position 3) and two conserved thymines and one conserved adenine are retained in the second half of the dyad at positions 3, 4, and 6, respectively [9], [10], [41]. There are also data to suggest that PhoP boxes may be located as many as 200–300 nucleotides upstream of the transcriptional start site and may be located in either orientation [8], [10].

Using these criteria, 35 of the 64 PhoP-regulated proteins detected in this study lacking a classic PhoP box contained such an atypical box, including those of well described PhoP-regulated proteins SlyB, PagC, and VirK [8]. The promoter regions for genes encoding two of the three Typhi-unique PhoP-regulated proteins detected in this study (CdtB and HlyE) also contained such boxes. We detected 29 proteins whose genes lack either a classic or atypical PhoP box, suggesting that PhoP may regulate their expression indirectly. For instance, we identified GroEL, a chaperone protein regulated by SlyA, which is itself a PhoP-regulated protein [16], [22], [42]. Similarly, PhoP directly up-regulates expression of PmrD, which then itself regulates the PmrAB operon [17]. Of interest, although ArnB (PmrH) is regulated through a PmrA binding site (a direct repeat of CTTAAT separated by 5 nucleotides) [43], we also found that the PmrA-regulated gene *arnB* also contains an atypical PhoP box, while the promoter of the gene for the protein Ugd detected in our analysis contains binding sites for PhoP, PmrA, and RcsB [44], [45]. These data suggest complex gene regulation.

PhoP is involved in intra-macrophage survival in *Salmonella*, and a number of the PhoP-regulated proteins detected in this study are involved in stress response, metabolism, and survival in limiting conditions (such as those that may be encountered within nutrient deficient macrophage vacuoles) [12], [13]. For instance, we detected Dps, a DNA-binding protein that protects DNA during stress; Daigle et al. also identified *dps* using selective capture of transcribed sequence (SCOTS) technology to analyze *S.* Typhi genes expressed within macrophages [26]. We similarly identified YbdQ, a universal stress protein, and PhoB a protein up-regulated in low phosphate conditions. Survival within macrophages may also involve the ability to withstand reactive oxygen and nitrogen species, and our identification of several oxidoreductases (STY3330, YghA) and thioredoxin reductase (TrxB) supports the observation of decreased intra-murine-macrophage survival of a *S.* Typhimurium mutant in *trxB* and the up-regulation of S. Typhi *trxB* mRNA within human macrophages [25], [46], [47]. The magnesium transporters MgtA and MgtB have previously been shown to be regulated in part by PhoP [4], and although we detected significant differential expression of MgtB in *S*. Typhimurium in PhoP-inducing compared to non-inducing concentrations of magnesium, our detection of differential expression of MgtA in *phoP^−^/Q^−^* mutants of *S.* Typhimurium and *S.* Typhi, and MgtB in a *phoP^−^/Q^−^* mutant of *S.* Typhi suggests involvement of other regulators in expression of these proteins.

Of the proteins identified in *S.* Typhimurium and not *S*. Typhi, two lacked corresponding gene sequences in *S.* Typhi: pSLT046, a putative carbonic anhydrase and part of a virulence plasmid in *S.* Typhimurium, and DkgA, involved in ascorbate biosynthesis and a pseudogene in *S*. Typhi. Of the proteins identified in *S*. Typhi and not *S*. Typhimurium, three (CdtB [STY1886], HlyE [STY1498], and STY1499) are of particular interest since corresponding genes are not found in *S*. Typhimurium, and these proteins may thus contribute to the pathogenicity of *S.* Typhi in humans. Our results are supported by a recent proteomic analysis by Ansong et. al. and Adkins et. al. which also showed that these proteins were expressed in PhoP-inducing conditions in a wild-type *S*. Typhi [20], [21]; and by comparative transcriptional analyses performed by Eriksson et. al. and Faucher et. al. which showed increased expression of these genes in *S.* Typhi recovered from human macrophages [23], [25].

CdtB (STY1886) is a homolog of the active subunit of a cytolethal distending toxin found in a number of bacterial pathogens including *Escherchia coli*, *Shigella dysenteriae*, *Haemophilus ducreyi, Actinobacillus actinomycetemcomitans,* and *Helicobacter hepaticus* [48]. CdtB expression is up-regulated intra-cellularly, and induces cell cycle arrest of host cells by causing DNA damage leading to distention of cells and enlargement of nuclei [48]. Interestingly, *S*. Typhi does not encode a homolog for the “B” subunit of the holotoxin, CdtA or CdtC, which mediates delivery of CdtB into target cells of the pathogens listed above [48]. However, Spano et al. have identified two proteins encoded within the same pathogenicity islet in *S*. Typhi, pertussis-like toxin A (PltA, STY1890) and pertussis-like toxin B (PltB, STY1891), that are induced intra-cellularly, form a complex with CdtB, and mediate its delivery into host cells [49]. Haghjoo and Galán have also previously shown that IgeR, which belongs to the DeoR family of transcription regulators, binds to the CdtB promoter and represses expression in extracellular bacteria [50]. Four lines of evidence support the hypothesis that PhoP may be involved in controlling CdtB expression of *S.* Typhi in macrophages. First, we detected significant CdtB protein and mRNA differential expression in PhoP-inducing versus non-inducing conditions. Second, we detected significant *pltA* and *pltB* mRNA differential expression in PhoP-inducing versus non-inducing conditions. Third, there was evidence for differential expression of the proteins PltA and PltB that approached statistical significance in wild type *S*. Typhi but not in the *phoP^−/^Q^−^* strain Ty800. Fourth, we identified an atypical PhoP box in the promoter region of CdtB.

The other two proteins we identified that are unique to *S*. Typhi and have no corresponding genes in *S*. Typhimurium are STY1499 and HlyE (STY1498). STY1499 is a secreted protein recently annotated TaiA (Typhi-associated invasin A) for its role in increasing bacterial uptake by macrophages [38]. It is co-transcribed with *hlyE*, also referred to as cytolysin A (ClyA) or SheA, which is a 34-kDa protein that forms pores in target cell membranes. Although *hlyE* is found in *S*. Typhi and *S*. Paratyphi A, it is absent from the genome of many other *Salmonella enterica* serovars including Paratyphi B, Paratyphi C, Typhimurium, Enteritidis, and others [51], [52]. HlyE shares >90% amino acid identity with ClyA in *E. coli* K-12, and ClyA has cytotoxic activity in murine and human macrophages [53]–[56]. In *S*. Typhi, HlyE contributes to cytotoxicity in epithelial cells, and affects bacterial growth within human macrophages [38]. In standard in vitro growth conditions, HlyE production is repressed in both *E. coli* and *S.* Typhi; however, humans infected with *S.* Typhi and *S*. Paratyphi A produce substantial levels of HlyE-specific antibodies [57]. Prior studies into the regulation of HlyE have shown that in *E. coli*, the global regulator H-NS (histone-like nucleoid-structuring protein) silences HlyE. This repression is antagonized by SlyA, which competes for binding at the H-NS promoter and allows cyclic AMP receptor protein (CRP) and fumarate and nitrate reduction regulator (FNR) to activate HlyE expression [54], [55]. The expression of *slyA* has been shown to be regulated by PhoP in *S.* Typhimurium, and SlyA itself regulates additional proteins, some co-regulated with PhoP, involved in virulence and survival within macrophages [42], [51], [58]–[60]. Although the PhoP regulation of SlyA has not previously been documented for *S.* Typhi, we did detect significant increase in *slyA* mRNA in PhoP-inducing conditions compared to non-inducing conditions. SlyA expression in *S.* Typhi increases upon infection of human macrophages and SlyA regulates *hlyE* expression in *S.* Typhi [42], [51], [58], [59]. DNA footprinting has confirmed the presence of a SlyA binding site (TTATATATTTAA) in the *hlyE* gene of *E. coli* located downstream of the transcription start site [52], and this binding site is also present in the *hlyE* gene of *S*. Typhi. In our analysis, we also found that the *hlyE* promoter contains an atypical PhoP box, supporting direct and indirect PhoP regulation of HlyE expression, an observation recently confirmed by Faucher *et al* [38].

Our study has a number of limitations. We were limited to comparing those proteins detected by current mass spectrometry techniques. More sensitive approaches could extend our findings. Two of our three wild type strains are also *rpoS* mutants (LT2 and Ty2). RpoS is an alternative sigma factor important for stationary phase survival and stress resistance. PhoP induces IraP, which protects RpoS from MviA-dependent degradation [19]. We attempted to limit any affect of the *rpoS* system by comparing Ty2 to its derivative Ty800, and LT2 to its derivative LT2 *phoP^−^/Q^−^*. In addition, of the proteins identified only in *rpoS* wild type *S*. Typhi strain CT18 and not in LT2 or Ty2, none are known to be RpoS dependent. In vitro growth of bacteria was also most rapid in wild type strains grown in media containing high magnesium and slowest in mutant strains grown in media containing low magnesium, and despite our harvesting of bacteria at mid-log for all cultures, such differential growth could explain in part our detection of repressed ribosomal proteins in PhoP-inducing conditions. However, our results are supported by gene expression profiling studies of *S.* Typhimurium infecting murine macrophages, in which 4 of the 9 ribosomal proteins identified as PhoP-repressed in our analysis were also repressed [23].

In conclusion, this is the first comparative protein expression profiling of the PhoP regulon of *Salmonella enterica* using strains of salmonella mutant in *phoP* and includes the first detailed proteomic analysis of any live attenuated bacterial candidate vaccine being tested in humans (Ty800). This study extends our understanding of the PhoP regulon in *S*. Typhi and *S*. Typhimurium, and has identified three Typhi-unique proteins (CdtB, HlyE and STY1499) that may be involved in human virulence and warrant further evaluation.

## Supporting Information

Table S1 Spectral counts and statistical analyses of identified PhoP-regulated proteins across *Salmonella* strains and growth conditions. List of the identified PhoP-regulated proteins in our analysis across *Salmonella* strains and growth conditions with raw spectral counts and p-values.
(0.04 MB XLS)

Table S2 PhoP up-regulated proteins identified in both *S.* Typhi and *S.* Typhimurium. List of PhoP up-regulated proteins identified in our analysis that are common to both *S.* Typhi and *S.* Typhimurium.
(0.09 MB DOC)

Table S3 PhoP-repressed proteins identified in both *S.* Typhi and *S.* Typhimurium. List of PhoP-repressed proteins identified in our analysis in both *S.* Typhi and *S.* Typhimurium.
(0.04 MB DOC)
