# Supplementary material for: Comparative Proteomic Analysis of the PhoP Regulon in Salmonella enterica Serovar Typhi Versus Typhimurium
Source: PLoS One. 2009 Sep 10;4(9):e6994. doi: 10.1371/journal.pone.0006994 (PMC2736619; doi:10.1371/journal.pone.0006994)
Supplement: Table S2 — PhoP up-regulated proteins identified in both S. Typhi and S. Typhimurium. List of PhoP up-regulated proteins identified in our analysis that are common to both S. Typhi and S. Typhimurium. (0.09 MB DOC) [file pone.0006994.s002.doc]

**Table S2: PhoP up-regulated proteins identified in both *S*. Typhi and *S***. Typhimurium

| **Functional Category** | **CT18 Locus** | **Ty2 Locus** | **LT2 Locus** | **Gene Name** | **Function** | **Ty2** | **CT18** | **LT2** |
| --- | --- | --- | --- | --- | --- | --- | --- | --- |
| **Pathogencity/Adaptation/ Chaperones** | STY0351 | t2544 | STM0306 | ‡ | probable outer membrane adhesin | X | X | X |
|  | STY1878 | t1119 | STM1246* | *pagC*‡ | outer membrane invasion protein | X | X | X |
|  | STY4690 | t4382 | STM4330 | *groEL* | TCP-1/cpn60 chaperonin family superfamily | X | X | X |
| **Regulators** | STY0432 | t2467 | STM0397 | *phoB*‡ | positive response regulator for pho regulon | X | X | X |
|  | STY0492 | t2410 | STM0450 | *lon*‡ | ATP-dependent protease La | X | X | X |
|  | STY1271 | t1689 | STM1231* | *phoP*† | transcriptional regulatory protein | X | X | X |
| **Membrane Surface Structures** | STY0468 | t2434 | STM0429 | *phnS* | putative 2-aminoethylphophonate-binding periplasmic protein precursor | X |  | X |
|  | STY0872 | t2055 | STM0833 | *ompX*‡ | outer membrane protein | X | X | X |
|  | STY0896 | t2033 | STM0863* | *dacC*‡ | D-alanyl-D-alanine carboxypeptidase; penicillin-binding protein 6a | X | X | X |
|  | STY1677 | t1313 | STM1445* | *slyB*‡ | lipoprotein SlyB, putative | X | X | X |
|  | STY2529 | t0564 | STM2299 | *arnA*‡ | bifunctional polymyxin resistance protein | X | X | X |
|  | STY3450 | t3187 | STM3267 |  | lipoprotein, putative | X | X | X |
|  | STY4231 | t3942 | STM3580 | ‡ | lipoprotein, putative | X | X | X |
|  | STY4254 | t3964 | STM3557 | *ugpB* | glycerol-3-phosphate periplasmic binding protein | X | X | X |
| **Central /Intermediary/Misc. Metabolism** | STY0456 | t2446 | STM0417 | *ribH* | riboflavin synthase subunit beta | X | X | X |
|  | STY0956 | t1976 | STM0958 | *trxB* | thioredoxin reductase | X | X | X |
|  | STY1155 | t1801 | STM1119 | *wrbA* | TrpR binding protein WrbA |  | X | X |
|  | STY1789 | t1202 | STM1322 |  | probable hydrolase | X |  | X |
|  | STY2289 | t0793 | STM2080* | *udg*† | UDP-glucose/GDP-mannose dehydrogenase | X | X | X |
|  | STY2527 | t0566 | STM2297* | *arnB*‡ | probable lipopolysaccharide biosynthesis protein | X | X | X |
|  | STY2666 | t0427 | STM2430* | *cysK* | cysteine synthase A | X | X | X |
|  | STY2711 | t0385 | STM2474* | *tktB* | transketolase |  | X | X |
|  | STY3330 | t3077 | STM3157 | *yghA* | oxidoreductase |  | X | X |
|  | STY3807 | t3555 | STM4064 | *cdh*‡ | CDP-diacylglycerol pyrophosphatase | X | X | X |
|  | STY3852 | t3595 | STM4026 | *yihX*‡ | hydrolase, haloacid dehalogenase-like family, putative | X | X | X |
|  | STY4355 | t4062 | STM3443 | *bfr* | bacterioferrin |  | X | X |
|  | STY4519 | t4225 | STM4319* | *phoN*‡ | nonspecific acid phosphatase precursor | X | X | X |
| **Energy Metabolism** | STY2370 | t0715 | STM2141 | *fbaB* | fructose-bisphosphate aldolase |  | X | X |
| **Degradation of Macro-molecules** | STY2849 | t2616 | STM2660 | *clpB*‡ | protein disaggregation chaperone | X | X | X |
|  | STY1334 | t1629 | STM1716 |  | sohB protein, peptidase U7 family VC1060 |  | X | X |
| **Phage/IS Elements** | STY2899 | t2675 | STM2781* | *virK*‡ | virulence protein | X | X | X |
|  | STY4721 | t4415 | STM4364 | *hflC*‡ | FtsH modulator |  | X | X |
| **Information Transfer** | STY0870 | t2057 | STM0831 | *dps*‡ | DNA protection during starvation conditions |  | X | X |
| **Conserved Hypothetical** | STY0662 | t2253 | STM0614 | *ybdQ*‡ | putative universal stress protein | X | X | X |

"X" represents significant differential detection of the protein in this strain (see text and Supplemental Table 1).

*Previously described PhoP-regulated gene.

†Promoter region contains a typical PhoP box defined as a dyad of (T/G)GTTTA separated by 5 nucleotides.

‡Presence of an atypical PhoP box defined as a dyad of (T/G)GTTTA separated by 5 nucleotides in the promoter region, allowing four substitutions as long as the following positions were conserved: a thymine in the first dyad half (at position 3) and two conserved thymines and one conserved adenine in the second dyad half at positions 3, 4, and 6, respectively, within 200-300 nucleotides of transcriptional start site (see text).
