# Supplementary material for: Comparative Proteomic Analysis of the PhoP Regulon in Salmonella enterica Serovar Typhi Versus Typhimurium
Source: PLoS One. 2009 Sep 10;4(9):e6994. doi: 10.1371/journal.pone.0006994 (PMC2736619; doi:10.1371/journal.pone.0006994)
Supplement: Table S3 — PhoP-repressed proteins identified in both S. Typhi and S. Typhimurium. List of PhoP-repressed proteins identified in our analysis in both S. Typhi and S. Typhimurium. (0.04 MB DOC) [file pone.0006994.s003.doc]

**Table S3: PhoP-repressed proteins identified in both *S*. Typhi and *S***. Typhimurium

| **Functional Category** | **CT18 Locus** | **Ty2 Locus** | **LT2**  **Locus** | **Gene Name** | **Function** | **Ty2** | **CT18** | **LT2** |
| --- | --- | --- | --- | --- | --- | --- | --- | --- |
| **Information transfer** | STY0981 | t1953 | STM0981 | *rpsA* | 30S ribosomal protein S1 | X | X | X |
|  | STY3735 | t3477 | STM4150 | *rplA*‡ | ribosomal protein L1 | X |  | X |
|  | STY4351 | t4058 | STM3447 | *rpsG* | ribosomal protein S7 | X |  | X |
|  | STY4352 | t4059 | STM3446 | *fusA* | elongation factor EF-2 | X |  | X |
|  | STY4361 | t4068 | STM3437 | *rplB* | ribosomal protein L2 | X | X | X |
|  | STY4364 | t4071 | STM3434 | *rpsC* | ribosomal protein S3 | X | X | X |
|  | STY4370 | t4077 | STM3428 | *rplE*‡ | ribosomal protein L5 | X | X | X |
|  | STY4373 | t4080 | STM3425 | *rplF* | ribosomal protein L6 | X |  | X |
|  | STY4375 | t4082 | STM3423 | *rpsE*‡ | 30S ribosomal protein S5 | X | X | X |

"X" represents significant differential detection of the protein in this strain (see text and Supplemental Table 1).

‡Presence of an atypical PhoP box defined as a dyad of (T/G)GTTTA separated by 5 nucleotides in the promoter region, allowing four substitutions as long as the following positions were conserved: a thymine in the first dyad half (at position 3) and two conserved thymines and one conserved adenine in the second dyad half at positions 3, 4, and 6, respectively, within 200-300 nucleotides of transscriptional start site (see text).
